# Supplementary material for: A Newly Incompatibility F Replicon Allele (FIB81) in Extensively Drug-Resistant Escherichia coli Isolated from Diseased Broilers
Source: Int J Mol Sci. 2024 Jul 30;25(15):8347. doi: 10.3390/ijms25158347 (PMC11312129; doi:10.3390/ijms25158347)
Supplement: Supplementary file 1 [file ijms-25-08347-s001.zip › Supplementary Figure.pdf]

```
               10        20        30        40        50        60        70        80        90       100
Query ATTCAGACATCAAAAAACTGTTCGGCGAGGTGGATAAGTCCTCCGGTGAGCTGGTGACACTGACACCAAACAATAACAACACCGTACAACCTGTGGCGAT
  Ref ..................................................................................................C.

              110       120       130       140       150       160       170       180       190       200
Query GATGCGTCTGGTTATTTTTGTACCGACCCTTAAATCACTGAAGAACAGTAAAAAAAATACACTGTCACGTACTGATGCCACGGAAGAGCTGACACGTCTT
  Ref ...........GCG.......................................................................................

              210       220       230       240       250       260       270       280       290       300
Query TCCCTGGCCCGTGCTGAGGGATTCGATAAGGTTGAGATCACCGGCCCCCGCCTGGATATGGATAATGATTTCAAGACCTGGGTGGGGATCATTCATTCCT
  Ref ....................................................................................................

              310       320       330       340       350       360       370
Query TTGCCCGCCATAACGTGATTGGTGACAAAGTTGAACTGCCTTTTGTTGAGTTTGCAAAACTGTGTGGTATACC
  Ref .........................................................................
```

Figure S1. Matching sequences of FIB-replicon alleles. The query sequence of the FIB replicon examined in plasmid extracts from *E. coli* (O125) was matched with FIB1 (373 nucleotides) reference sequence in www.pubmlst.org/plasmid/.database. Four nucleotides C, G, C, and G in FIB1 allele at positions 99, 112, 113, and 114 were substituted with A, T, T, and A respectively in the query sequence. These point mutations referred to a new FIB replicon, which was identified as FIB81 allele (accession number OR453940).
