# Supplementary material for: A Newly Incompatibility F Replicon Allele (FIB81) in Extensively Drug-Resistant Escherichia coli Isolated from Diseased Broilers
Source: Int J Mol Sci. 2024 Jul 30;25(15):8347. doi: 10.3390/ijms25158347 (PMC11312129; doi:10.3390/ijms25158347)
Supplement: Supplementary file 1 [file ijms-25-08347-s001.zip › Supplementary Tables.pdf]

Table S1: Resistance patterns of 31 *E. coli* strains isolated from broilers

Strain No.	Resistance pattern	No. of AMA	MAR index
1-3	AMP, SAM, AMC, CRO, CFP, CTX, DO, CIP, CN, C, AZM, SXT, FOS, CT	14	1
4	AMP, SAM, AMC, CRO, CFP, CTX, DO, CIP, C, AZM, SXT, FOS, CT	13	0.93
5	AMP, SAM, AMC, CRO, CFP, CTX, DO, CIP, CN, C, SXT, FOS, CT	13	0.93
6	AMP, SAM, AMC, CRO, CTX, DO, CIP, CN, C, AZM, SXT, FOS, CT	13	0.93
7	AMP, SAM, AMC, CRO, CTX, DO, CIP, C, AZM, SXT, FOS, CT	12	0.86
8	AMP, SAM, AMC, CRO, CTX, DO, CIP, C, AZM, SXT, FOS, CT	12	0.86
9	AMP, SAM, AMC, CRO, CFP, CTX, DO, CIP, CN, C, SXT, CT	12	0.86
10	AMP, SAM, AMC, CRO, CFP, CTX, DO, CIP, CN, AZM, FOS, CT	12	0.86
11-14	AMP, SAM, AMC, CRO, CTX, CIP, CN, C, AZM, SXT, FOS, CT	12	0.86
15	AMP, SAM, AMC, CRO, CTX, DO, CIP, CN, C, AZM, SXT, CT	12	0.86
16	AMP, SAM, AMC, CTX, CIP, AZM, SXT, FOS, CT	9	0.64
17	AMP, SAM, AMC, CTX, DO, AZM, CT	7	0.5
18	AMP, SAM, AMC, CFP, DO, CT	6	0.43
19	AMP, SAM, DO, CN, AZM, CT	6	0.43
20	AMP, SAM, CN, AZM, FOS, CT	6	0.43
21	AMP, SAM, AZM, SXT, CT	5	0.36
22	AMP, SAM, AMC, DO, CT	5	0.36
23-24	AMP, SAM, CRO, CT	5	0.36
25	AMP, SAM, AMC, CT	4	0.29
26	AMP, CT, CTX, CIP	4	0.29
27-31	Pan-susceptible	0	0

AMP: ampicillin, SAM: ampicillin/sulbactam, AMC: amoxicillin/ clavulanate, CRO: ceftriaxone, CFP: cefoperazone, CTX: cefotaxime, DO: doxycycline, CIP: ciprofloxacin, CN: gentamycin, C: chloramphenicol, AZM: azithromycin, SXT: sulphamethoxazole/trimethoprim, FOS: fosfomycin and CT: colistin. AMA: antimicrobial agent, MAR: multiple antimicrobial resistance. Underlined strains are those selected for incF replicon typing.

Table S2: Resistance patterns of 36 *Salmonella spp.* strains isolated from broilers

Strain No.	Resistance pattern	No. of AMA	MAR index
1	AMP, SAM, AMC, CRO, CFP, CTX, DO, CIP, CN, C, AZM, SXT, FOS, CT	14	1
2	AMP, SAM, AMC, CRO, CFP, CTX, DO, CIP, CN, C, AZM, SXT, CT	13	0.93
3	AMP, SAM, AMC, CRO, CTX, CIP, CN, C, AZM, SXT, FOS, CT	12	0.86
4	AMP, SAM, AMC, CRO, CFP, CTX, DO, CIP, C, SXT, FOS, CT	12	0.86
5	AMP, SAM, AMC, CRO, CFP, CTX, DO, CIP, CN, C, SXT, CT	12	0.86
6	AMP, SAM, AMC, CFP, CTX, DO, CIP, C, AZM, SXT, CT	11	0.79
7	AMP, SAM, AMC, CTX, DO, CIP, CN, C, AZM, SXT, CT	11	0.79
8	AMP, SAM, AMC, CRO, CFP, CTX, DO, CN, C, AZM, CT	11	0.79
9	AMP, SAM, AMC, CRO, CTX, DO, CIP, C, SXT, CT	10	0.71
10	AMP, SAM, AMC, CTX, DO, CN, C, SXT, CT	9	0.64
11	AMP, SAM, AMC, CRO, CFP, DO, CT	7	0.5
12-14	AMP, SAM, AMC, CRO, CTX, AZM, CT	7	0.5
15	AMP, SAM, AMC, CFP, DO, CT	6	0.43
16	AMP, SAM, AMC, CFP, CN, CT	6	0.43
17	AMP, SAM, AMC, CFP, SXT, CT	6	0.43
18	AMP, SAM, CFP, CIP, CN, CT	6	0.43
19	AMP, SAM, AMC, C, SXT, CT	5	0.36
20-21	AMP, SAM, AMC, CN, CT	5	0.36
22	AMP, SAM, CRO, SXT, CT	5	0.36
23	AMP, SAM, AMC, CN, CT	5	0.36
24	AMP, SAM, CFP, SXT, CT	5	0.36
25	AMP, SAM, CRO, DO, CT	5	0.36
26	AMP, SAM, CFP, CIP, CT	5	0.36
27-29	AMP, SAM, AMC, CT	4	0.29
30	AMP, SAM, DO, CT	4	0.29
31	AMP, SAM, CRO, CT	4	0.29
32-36	Pan-susceptible	0	0

AMP: ampicillin, SAM: ampicillin/sulbactam, AMC: amoxicillin/ clavulanate, CRO: ceftriaxone, CFP: cefoperazone, CTX: cefotaxime, DO: doxycycline, CIP: ciprofloxacin, CN: gentamycin, C: chloramphenicol, AZM: azithromycin, SXT: sulphamethoxazole/trimethoprim, FOS: fosfomycin and CT: colistin. AMA: antimicrobial agent, MAR: multiple antimicrobial resistance. Underlined strains are those selected for incF replicon typing.
